# Examining the Use of HIV Self-Testing to Support PrEP Delivery: a Systematic Literature Review

**DOI:** 10.1007/s11904-022-00617-x

**Published:** 2022-07-29

**Authors:** Catherine Kiptinness, Alexandra P. Kuo, Adriana M. Reedy, Cheryl C. Johnson, Kenneth Ngure, Anjuli D. Wagner, Katrina F. Ortblad

**Affiliations:** 1 Department of Public and Global Health, University of Nairobi, Nairobi, Kenya; 2 Department of Pharmacy, University of Washington, Seattle, WA 98195 USA; 3 Public Health Sciences Division, Fred Hutchinson Cancer Center, 1100 Fairview Ave N, Seattle, WA 98109 USA; 4 Global HIV, Hepatitis and STI Programmes, World Health Organization, Geneva, Switzerland; 5 Department of Community Health, Jomo Kenyatta University of Agriculture and Technology, Nairobi, Kenya; 6 Department of Global Health, University of Washington, Seattle, WA 98105 USA

**Keywords:** Systematic literature review, HIV self-testing, PrEP delivery, HIV prevention, Implementation science, Sub-Saharan Africa

## Abstract

**Purpose of Review:**

HIV self-testing (HIVST) has the potential to expand access to and uptake of HIV pre-exposure prophylaxis (PrEP) delivery. We conducted a systematic literature review to understand the evidence on HIVST use for PrEP delivery.

**Recent Findings:**

After screening 1055 records, we included eight: three randomized trials and five values and preferences studies. None measured PrEP initiation. Most studies occurred in Sub-Saharan Africa (7/8) and included different populations. One trial found that HIVST use between quarterly clinic visits as part of an adherence package with biofeedback slightly increased adherence; the other two trials found that HIVST use between or in lieu of quarterly clinic visits had no significant or non-inferior effects on adherence. HIVST to support PrEP delivery was acceptable, feasible, and preferred.

**Summary:**

HIVST use for PrEP continuation largely resulted in similar outcomes to standard-of-care delivery and was perceived acceptable and feasible. Further research is needed to optimize HIVST use within PrEP programming.

**Supplementary Information:**

The online version contains supplementary material available at 10.1007/s11904-022-00617-x.

## Introduction

Pre-exposure prophylaxis (PrEP) use has been increasing steadily since it was first recommended by the World Health Organization (WHO) for HIV prevention in 2015 [1]; however, many individuals that could benefit from PrEP lack access [2]. Common barriers to PrEP initiation and continuation in high HIV prevalence settings include long wait times at often overcrowded healthcare facilities [3] and the need for frequent clinic visits for HIV testing and PrEP refills which lead to high client opportunity costs [4], as well as stigma associated with PrEP access and use [5]. Simplified and novel models of PrEP delivery are needed to increase the reach and access of PrEP to populations at HIV risk not currently engaged in PrEP care [6].

HIV self-testing (HIVST), which has been recommended as an effective HIV testing approach since 2016, has the potential to simplify and support PrEP delivery [7]. Currently, the WHO recommends that individuals using PrEP should test for HIV every 3 months to detect potential breakthrough infections [8], and that HIVST can be used as a way to maintain PrEP programs in the context of the COVID-19 pandemic [9]. Currently, PrEP initiation is generally linked to clinic-based HIV testing services, followed by quarterly testing that accompanies PrEP refills. HIVST could enable individuals taking PrEP to test routinely, by replacing or complementing existing testing intervals with providers, which could potentially increase access and adherence to PrEP services [10]. Additionally, HIVST could enable greater differentiated service delivery for PrEP initiation and continuation in new settings beyond the bounds of traditional healthcare facilities, such as at private pharmacies or during at-home visits [11].

We sought to understand the existing evidence on the use of HIVST to support PrEP delivery to inform policy making. In this review, we specifically sought to understand how HIVST has been used to support PrEP initiation and continuation by identifying studies that measured the effectiveness of, values and preferences for, and economic outcomes associated with these interventions.

## Methods

We followed guidelines from the Cochrane Collaboration [12] and PRISMA [13] for the completion of this systematic literature review. Our review protocol is included in Appendix 1 (Supplemental material) and published on PROSPERO (ID CRD42022296937).

### Search Strategy

We searched eleven electronic databases (ClinicalTrials.gov, Web of Science, Global Health Database, Cochrane Central Register of Controlled Trials, Global Index Medicus, WHO ICTRP trials, APA PsychInfo, Social Services Abstract, PubMed, CINAHL, and EMBASE) through 25 August 2021 for peer-reviewed articles. To search these databases, we used the following primary terms: (“Pre-Exposure Prophylaxis” [Medical Subjects Heading (MeSH)] OR “pre-exposure prophylaxis” [title and abstract (tiab)] OR “preexposure prophylaxis” [tiab] OR “antiretroviral prophylaxis” [tiab] OR “preexposure chemoprophylaxis” [tiab] OR PrEP [tiab]), developed in collaboration with a librarian at the University of Washington (Seattle, USA).

Since both HIVST and PrEP are relatively new interventions, to capture any ongoing studies we additionally searched the following trial registrations (up to 25 August 2021) using the terms “HIV testing” AND “self”/”home”/”unsupervised” AND “pre-exposure prophylaxis”: ClinicalTrials.gov, the WHO International Clinical Trials Registry Platform, the Pan-African Clinical Trials Registry, and the Australian New Zealand Clinical Trials Registry. Additionally, we searched abstracts using the same search terms from the following conferences (also up to 25 August 2021): International AIDS Conference (AIDS), International AIDS Society Conference on HIV Science (IAS), and Retroviruses and Opportunistic Infections (CROI), and HIV Research for Prevention (HIVR4P).

The protocol was developed in coordination with the WHO team (CJ and other non-authors). We included no geographic restrictions on our database, trial registry, or abstract searches, and only included English publications and peer-reviewed papers. We reviewed the reference list of included studies for additional publications and contacted experts in the field to identify any additional articles not found through other search methods. We continued to search for trials we found that did not have outcomes reported until a month before we submitted this manuscript for publication and added any identified study results to the review.

### Study Selection

We included studies for extraction that focused on the use of HIVST for PrEP initiation or continuation. This included studies that used HIVST in lieu of traditional HIV testing services (HTS) as well as in addition to HTS at different time points. We defined PrEP initiation as the process of being prescribed and dispensed PrEP for the first time or after a long break (i.e., restarting PrEP). We defined PrEP continuation as continuing PrEP use following initiation, which included measurements of retention and drug refills as well as adherence (e.g., self-reported PrEP use, detectable drug levels in blood/hair/urine, pill counts). We identified three potential types of PrEP continuation: (1) *as prescribed* (e.g., often every 3 months, could be linked or not necessarily linked to PrEP refills), (2) *as needed* (e.g., for stopping and restarting as periods of HIV risk change), and (3) *as desired* (e.g., between PrEP visits and refills). Studies that used HIVST to support any of these types of PrEP continuation were included in our review.

Additionally, we only included studies for extraction that measured or planned to measure effectiveness, values and preferences, or economic outcomes related to HIVST use for PrEP delivery. Studies that measured effectiveness outcomes directly compared a group, facility, geographic area, or population that used HIVST to a group, facility, geographic area, or population that used only standard HTS without HIVST for PrEP delivery. These studies also included at least one of the primary outcomes: (1) PrEP initiation or (2) PrEP continuation. If studies met all the criteria but did not present comparative data, we still included them and presented them as case studies. Studies that measured values and preferences outcomes included qualitative or quantitative data on client and provider opinions, perspectives, values, and preferences related to PrEP initiation and continuation supported with the use of HIVST at clinics or in the community. Finally, studies that measured economic outcomes presented primary data on the costs, cost-effectiveness, cost-utility, or cost-benefit of using HIVST to support PrEP initiation or continuation.

Two authors (AK and CK) reviewed all titles and abstracts to ensure reliable application of the inclusion criteria. Authors AK and AR independently screened all studies at the full-text level and noted reasons for exclusion. We resolved any disagreements on inclusion via group consensus following discussion. We used Covidence (Melbourne, Australia) to collaboratively screen titles/abstracts and facilitate the full-text review.

### Data Extraction and Analysis

We collaboratively developed a structured data abstraction form in Covidence. Thereafter, two authors (AK and AR) extracted the following information from each study: author; title; year; study location; study design; study population (e.g., men who have sex with men); sample size; a description of the intervention (e.g., HIVST for PrEP initiation or continuation); a description of the comparison group (if available); and a summary of key findings. For effectiveness outcomes, we included effect size estimates, confidence intervals, and significance level. For values and preference outcomes, we included a descriptive summary of key findings. Authors AK and AR assessed the risk of bias for each study according to guidance by the Cochrane Collaboration and used a risk-of-bias visualization (robvis) tool [14] to create figures that reflect these outcomes (Appendix Figs. 1–4). To resolve any differences in data extraction and risk of bias assessment, we reviewed any disputed studies together until we achieved total group consensus (AK, AR, AW, CJ, CK, KN, and KO).

We summarized the key findings of the review using a modified Developing and Evaluating Communication Strategies for Support Informed Decisions and Practice Based on Evidence (DECIDE) Evidence to Decision (EtD) framework to summarize our results. The DECIDE EtD framework includes criteria that have been determined as necessary for informing evidence-based recommendations to key stakeholders such as health professionals, policymakers, patients, and the general public [15, 16].

## Results

**Fig. 1:**
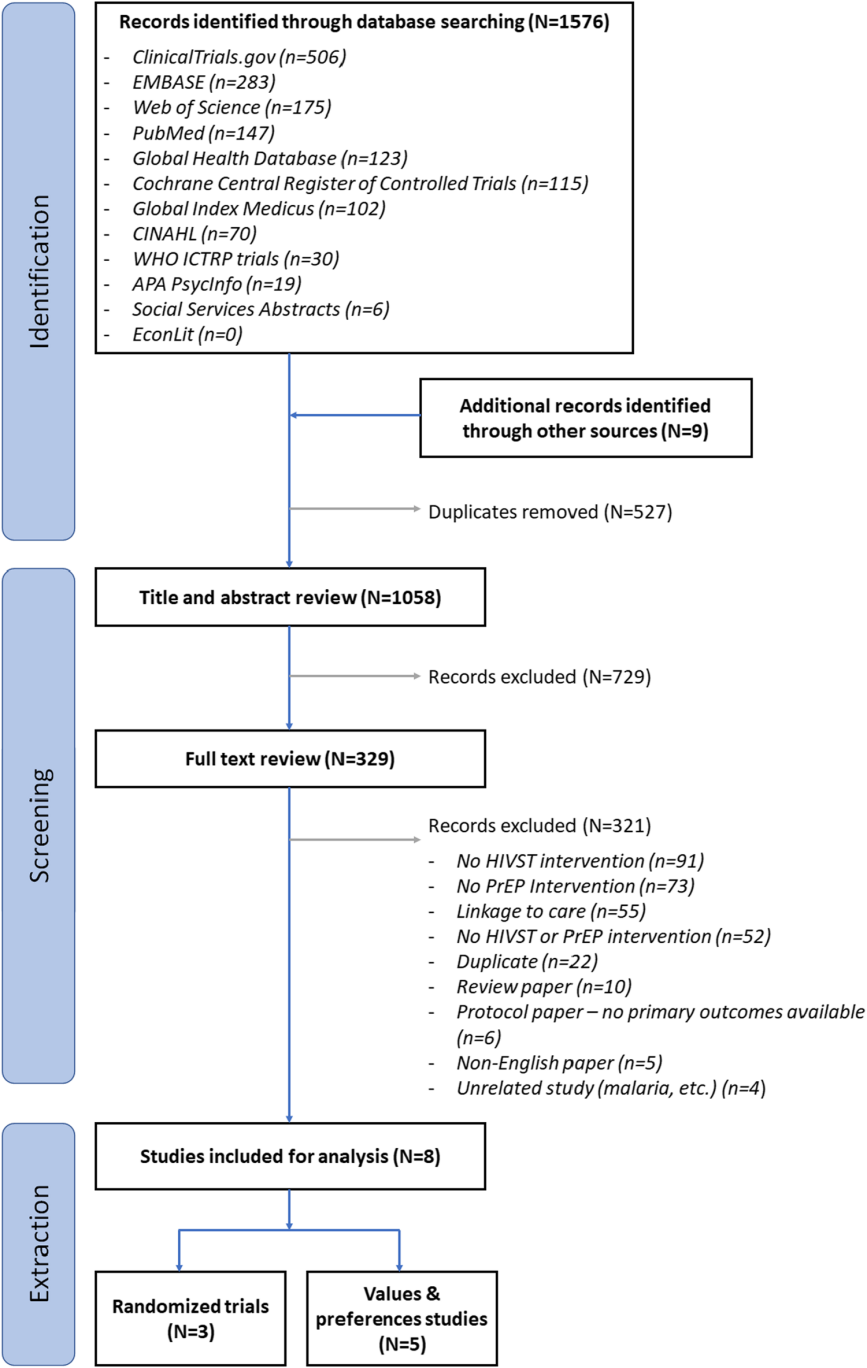
PRISMA diagram of reviewed and included studies in our review of the literature on HIV self-testing to support HIV pre-exposure prophylaxis delivery

Our search yielded 1055 unique studies and after screening titles, abstracts, and full manuscripts, we identified eight for inclusion: three randomized controlled trials (RCTs) measuring effectiveness outcomes and five studies measuring values and preferences (Fig. 1). We identified two of the three included RCTs in December 2021, four months after completion of the initial review in August 2021, while reviewing relevant study protocols identified in the initial review (Appendix Table 1).

We describe the characteristics of the studies included in Table 1. The majority of studies were in sub-Saharan Africa (86%) and in different populations (e.g., transgender people, sex workers, HIV serodifferent couples). All included studies focused on HIVST-supported models for daily, oral PrEP delivery, and used a combination of both oral-fluid and blood-based HIVST. All the RCTs reported the effect of these models on PrEP continuation; no studies reported on the effect of HIVST-supported PrEP delivery on initiation. Among the studies that reported on HIVST use for PrEP continuation, four (50%) reported on continuation as desired (with HIVST for testing between refill visits) and six (75%) reported on PrEP continuation as prescribed (with HIVST for refilling). Like PrEP initiation, no studies reported HIVST use for PrEP continuation as needed (with HIVST to inform stopping and restarting PrEP).

**Table 1:** Descriptive characteristics of studies included in our review, *N*=8^1^

Characteristics	Randomized controlled trials	Values and preferences studies
	*N*=3 (38%)^2^	*N*=5 (63%)^2^
WHO region		
African Region (AFR)	3 (100%)	4 (80%)
Region of the Americas (AMR)	0	1 (20%)
Country income level		
Low and lower-middle	3 (100%)	4 (80%)
Upper-middle	0	1 (20%)
Study population^3^		
Adolescent girls and young women	0	1 (20%)
HIV serodifferent heterosexual couples	1 (33%)	1 (20%)
Men who have sex with men	0	1 (20%)
Postpartum women	1 (33%)	0
Sex workers	1 (33%)	3 (60%)
Transgender people	1 (33%)	2 (40%)
Women not in HIV serodifferent couples	1 (33%)	1 (20%)
Distribution of PrEP		
Facility/clinic	3 (100%)	4 (80%)
Home-based	0	1 (20%)
PrEP modality		
Daily oral	3 (100%)	5 (100%)
HIVST type ^3^		
Blood-based	1 (33%)	1 (20%)
Oral-fluid based	3 (100%)	4 (80%)
Not mentioned	0	1 (20%)
Primary outcome: HIVST for PrEP^3^		
Continuation as desired *(e.g., between visits/refills)*	1 (33%)	3 (60%)
Continuation as prescribed *(e.g., often for refills)*	3 (100%)	3 (60%)
Continuation as needed *(e.g., for stopping and restarting)*	0	0
Initiation	0	0

Abbreviations: *WHO*, World Health Organization; *PrEP*, pre-exposure prophylaxis; *HIV*, human immunodeficiency virus; *STI*, sexually transmitted infections

^1^Data are shown for eight included manuscripts and conference abstracts, which represented seven unique PrEP trials, delivery programs, or values and preferences studies (because some studies reported findings for the same population or the same PrEP program)

^2^There was no overlap in randomized trials and values and preferences studies

^3^The frequencies and percentages in this category sum to greater than the total number of studies because several studies included more than one study population

### Effectiveness Outcomes

In the three RCTs that measured effectiveness outcomes, HIV self-tests were used in a unique way to support PrEP continuation (Table 2). The Empower study used oral-fluid HIV self-tests to support as-desired interim testing between quarterly clinic-based PrEP visits for sex workers in Uganda [17••]. The JiPime-JiPrEP study used both oral-fluid and blood-based HIV self-tests to support semiannual clinic-based PrEP visits with six-month PrEP dispensing and HIVST for as-prescribed interim testing at three months for HIV serodifferent couples and women singly enrolled in Kenya [18••]. The PrEP-PP study delivered oral-fluid HIV self-tests as part of a PrEP adherence promotion package with counseling and enhanced adherence biofeedback to support as-desired interim testing between quarterly clinic-based PrEP visits for postpartum women in South Africa [19••].

**Table 2:** Description of effectiveness studies and key findings related to HIVST use for PrEP delivery, *N*=3^1^

Study	Country, enrollment period	Population	Design	Intervention and outcome definition	Comparison	Effect on PrEP initiation and/or continuation	Other findings (e.g., retention, HIV incidence, creatinine levels)
**Randomized controlled trials (*N*=3)**							
Mujugira A, et al., *J Acquir Immune Defic Syndr* 2022 (NCT03426670) **Empower Study**	Uganda May 2018–Jan 2020	Sex workers *N*=110 *FSWs (N= 84); TGW (N=14); MSM (N=10); TGM (N=2)*	1:1 randomized trial (parallel assignment)	Intervention: Quarterly clinic-based PrEP visits with RDT and four HIV self-tests for monthly interim testing. Primary outcome: PrEP adherence at 12 months; as measured by electronic adherence monitoring (EAM, i.e., % days active device opened) and intracellular TFV-DP levels (any detection) in DBS samples.	SOC: Quarterly clinic-based PrEP visits with RDT.	Continuation: *Retention.* At 12 months, 81% in the intervention and 70% in the SOC (*p*=0.19) were retained in PrEP care. *Adherence.* At 12 months, 46% in the intervention and 54% in the SOC were adherent, as measured by EAM (aOR 3.32, 95% CI −5.3, 11.9). Then, 47% in the intervention and 53% in the SOC were adherent, as measured by DBS TFV-DP (aOR 1.01, 95% CI 0.91, 1.97)	HIVST use: Nearly all participants (99%) used ≥1 HIVST kit. HIV incidence: One seroconversion occurred during observation (0.9/100 person-years).
Ngure K, et al., *Lancet HIV* 2022 (NCT03593629) **JiPime-JiPrEP Trial**	Kenya May 2018–May 2021	People at HIV risk *N*=495 *Men (N=165) and women (N=130) in HIV SD couples; women not in SD couples (N=200)*	2:1 randomized non-inferiority trial (parallel assignment)	Intervention: 6-month PrEP dispensing supported with two interim HIVST (at 3 months) and biannual clinic visits with RDT. Primary outcomes: HIV testing (between enrollment and follow-up), PrEP refilling, and PrEP adherence (as measured by intracellular TFV-DP levels in DBS samples) at 6 months.	SOC: 3-month PrEP dispensing and quarterly clinic visits with RDT.	Continuation: *PrEP refilling.* At 6 months, 78% (257/329) in the intervention refilled PrEP, compared to 81% (134/166) in the SOC (RD −2·6%, 1-sided 95% CI lower bound -8.9%). *Adherence.* At 6 months, 61% (200/329) in the intervention were adherent compared to 57% (95/166) in the SOC (RD 2.4%, one-sided 95% CI lower bound −5.1). In a sub-group of single women, 51% (68/133) were adherent compared to 31% (21/67) in the SOC (RD 19.8%, 95% CI 5.8, 33.7)	HIV testing: At 6 months, 83% (274/329) of participants in the combined intervention arm had tested for HIV compared to 84% (140/166) of participants in the SOC arm (RD −1.2%, one-sided 95% CI lower bound −6.9) HIV incidence: At 6 months, no participants tested HIV positive during study implementation.
Davey DJ, et al., *Open Forum Infect Dis* 2022 (NCT04897737) **PrEP-PP**	South Africa Aug 2020–Apr 2021	Women, 1-6 months postpartum *N*=106	1:1 randomized trial (parallel assignment)	Intervention: A PrEP adherence promotion package: two HIVST kits delivered to women and their partners with counseling and enhanced adherence biofeedback through urine tenofovir testing. Primary outcomes: PrEP adherence (past 48–72 h) via urine TFV testing (any prevalence) and partner HIV testing 1-month post randomization.	SOC: Clinic-based PrEP adherence counseling without biofeedback; referral for partner to attend facility for testing.	Continuation: *Adherence.* At 1 month, 62% (33/53) of women in the intervention had TFV in their urine compared to 34% (18/53) in SOC (RR 1.83, 95% CI 1.19, 2.82).	Partner HIV testing: Two-thirds (66%, 35/53) of women in the intervention reported that her partner tested for HIV compared to 17% (9/53) in SOC (RR 3.89, 95% CI 2.08, 7.27). HIV incidence: In each arm, 1 partner tested positive for HIV (8.6% positivity rate for intervention; 11% positivity rate for SOC).

Abbreviations: *PrEP*, pre-exposure prophylaxis; *HIV*, human immunodeficiency virus; *HIVST*, HIV self-test; FSWs, female sex workers; *MSM*, men who have sex with men; *TGW*, transgender women; *TGM*, transgender men; *SD*, serodifferent; *AGYW*, adolescent girls and young women; *SOC*, standard of care; *RDT*, rapid diagnostic testing; *DBS*, dried blood spots; *TFV-DP*, tenofovir-diphosphate; *RD*, risk difference; *OR*, odds ratio; *aOR*, adjusted odds ratio; *RR*, risk ratio; *CI*, confidence interval

^1^This section of the table reports findings from three included articles from randomized trials that had a comparator group

In the identified protocols for ongoing RCTs and case studies (Appendix Table 1–2), HIV self-tests were additionally used for continuation as prescribed by testing while waiting for clinic-based PrEP refills in Kenya [20] and continuation as desired by testing between quarterly clinic visits in Uganda [21] and Kenya [22].

#### Adherence

All three RCTs reported on the effect of HIVST-supported PrEP delivery models on PrEP adherence, as measured by electronic adherence monitoring (EAM) (Empower) [17••], intracellular tenofovir-diphosphate (TFV-DP) levels in dried bloodspot samples (Empower and JiPime-JiPrEP) [17••, 18••], and urine TFV testing (PrEP-PP) [19••]. The Empower study found that the delivery of HIV self-tests at PrEP clinic visits for as-desired testing between visits did not affect PrEP adherence at 12 months (EAM HIVST: 46% adherence; TFV-DP HIVST: 47% adherence) compared to no delivery of HIV self-tests at PrEP clinic visits (EAM standard-of-care: 54% adherence; TFV-DP standard-of-care: 53% adherence); EAM adjusted odd ratio [aOR] 3.32, 95% CI −5.3, 11.9; TFV-DP aOR 1.01, 95% CI 0.91, 1.97) [17••]. The PrEP-PP study found that the delivery of HIV self-tests, as part of a PrEP adherence support package that additionally included counseling and biofeedback for as-desired testing between clinic visits, did increase PrEP adherence at one month (62% adherence) compared to counseling with no HIV self-test delivery and biofeedback (34% adherence; risk ratio [RR] 1.83, 95% confidence interval [CI] 1.19, 2.82) [19••]. And finally, the JiPime-JiPrEP trial found that six-month PrEP dispensing supported interim as-prescribed HIVST at three months and resulted in non-inferior PrEP adherence (61% adherence) compared to three-month PrEP dispensing with clinic-based rapid diagnostic tests (RDT) at six months (57% adherence; risk difference [RD] 2.4%, one-sided 95% CI lower bound −5.1%) [18].

#### Retention and Refilling

The Empower study and JiPime-JiPrEP trial also measured the effect of HIVST-supported PrEP delivery models on PrEP retention and refilling, respectively [17••, 18••]. The Empower study found no effect of HIV self-test delivery at quarterly clinic-based PrEP visits on retention at 12 months [17••], while the JiPime-JiPrEP trial found that HIVST for interim testing between biannual clinic-based PrEP visits was non-inferior compared to quarterly clinic-based HIV testing at six months [18••].

#### HIV Testing Outcomes

All included RCTs had slightly different HIV testing outcomes. In the Empower study, almost all participants (99%) that received the intervention used more than one HIV self-test between PrEP clinic visits by 12 months [17••]. In the JiPime-JiPrEP trial, any HIV testing in the past six months was high (>80%) among participants that received six-month PrEP dispensing supported with interim HIVST and non-inferior compared to quarterly clinic-based HIV testing at six months [18••]. In the PrEP-PP study, HIV self-test delivery with PrEP adherence counseling increased partner testing in the past month compared to adherence without HIV self-test delivery at one month [19••].

#### HIV Incidence

In all three RCTs, HIV incidence was very low; only one participant seroconverted in the Empower study [17••] and no participants seroconverted in the JiPime-JiPrEP study [18••] and in the PrEP-PP study [19••]. None of the RCTs were powered to measure this outcome.

### Value and Preferences Outcomes

In the five studies that measured values and preference outcomes, the models of HIVST-supported PrEP delivery were all unique (Table 3). The Hoagland et al. study explored the potential for PrEP teleconsultation, supported with home delivery of PrEP and HIVST among men that have sex with men and transgender people in Brazil [23••]. The Partners Demonstration Project sub-study explored a very similar model as the Empower study [17••] (described above): HIV self-test delivery at quarterly PrEP clinic visits to support as-desired testing between visits among HIV serodifferent couples in Kenya [24•]. The Ortblad et al. study explored preferences for HIVST versus clinic-based testing for PrEP delivery among female sex workers in Uganda and Zambia [25•]. And the POWER study explored revealed preferences among adolescent girls and young women for HIVST versus provider-initiated HIV testing for PrEP continuation at family planning clinics in Kenya [26•].

**Table 3:** Description of studies exploring values and preferences for community-based PrEP service delivery, *N*=5^1^

Study	Country, enrollment period	Population	Study design	Intervention and outcome assessment	Key findings on preferences for HIVST-supported PrEP delivery
Hoagland, B. *et al. Braz J Infect Dis. 2021*	Brazil, April–May 2020	MSM, TGNB currently using PrEP *N*=680	Cross-sectional web-based survey	Intervention: PrEP teleconsultation, supported with home delivery of PrEP and HIVST. Values and preferences: Participants were asked how they would feel about PrEP teleconsultation (5-pt comfort Likert scale, top two responses indicated acceptability). They were also asked if they would prefer receiving PrEP refills + HIV self-test at home versus collecting them at the facility, and their awareness of, previous use, and willingness to use HIVST.	Acceptability of PrEP teleconsultation: The majority (70%, 373/534) of participant found PrEP teleconsultation acceptable. *• Acceptability of home-delivered PrEP.* The majority of respondents (87%, 593/680) reported preferring PrEP + HIVST home delivered instead of collecting PrEP at the facility. *• Acceptability of HIVST:* Awareness and acceptability of HIVST was high among participants. Most participants (79%, 391/495) were willing to use HIVST and 32% (218/680) received an HIV self-test during the social distancing period of the COVID pandemic.
Mujugira, *et al., J Int AIDS Soc* 2021 (NCT03426670) **Empower Study**	Uganda, June 2018–Jan 2020	SW, TGW, MSM, intimate partners SW (*N*=30): FSW (*N*=21); TGW (*N*=6); MSM (*N*=3) Intimate partners (*N*=10) of: FSW (*n*=4); TGW (*n*=5); MSM (*n*=1)	Qualitative study	Intervention: Four HIV self-tests delivered at each PrEP clinic visits (2 for their own use and 2 for testing sexual partners) for testing between quarterly clinic visits. Values and preferences: Participants and their sexual partners completed in-depth interviews (IDIs) that explored their experiences with HIVST, how HIVST was performed with sexual partners, the impact of HIVST on PrEP pill taking, HIV status disclosure and sexual risk behaviors after HIVST.	Overall finding: HIVST to support PrEP delivery/use was empowering for Ugandan SWs and their partners. Three types of empowerment were observed: (a) economic; (b) relational; and (c) sexual health. *• Economic empowerment:* HIVST-supported PrEP helps eliminate unknown client HIV status as a barrier to the provision of condomless sex with clients (associated with higher client fees). *• Relational empowerment:* HIVST restored trust in partners’ fidelity upon being reunited after a separation. This trust, in combination with condomless sex made possible by PrEP use, restored intimacy, empowering partnered relationships. *• Empowerment of sexual health:* HIVST-supported PrEP enabled SWs to take control of their HIV prevention efforts and avoid the stigma of public clinic visits.
Ngure K, et al., *J Int AIDS Soc*, 2017 (NCT02775929) **Partners Demonstration Project, sub-study**	Kenya, Nov 2013–June 2015	HIV uninfected partners *N*=222 Male (*N*=177) Female (*N*=45)	Case study	Intervention: Two HIV self-tests delivered for testing between quarterly clinic PrEP visits. Participants were recommended to self-test every time they started a new PrEP bottle. Values and preferences assessment: *Acceptability* of HIVST-supported PrEP continuation assessed qualitatively with IDIs and focus group discussions among participants that received the intervention. *Preferences* for oral vs. finger prick and self- vs. clinic-based testing assessed at quarterly clinic visits. *Feasibility* assessed quantitatively at these visits with participant self-reports on the number of self-tests performed, ease of performing the self-test, sharing of HIVST kits and results, and use of the 24-h helpline	Acceptability: HIVST between PrEP clinic visits reduced the anxiety associated participants experienced with waiting to return for scheduled clinic-based HIV testing. Preferences: Roughly half (52%) of participants reported they would prefer HIVST only for PrEP continuation, while some (39%) reported that they would prefer a mixture of HIVST and provider testing. Feasibility*:* Almost all (93%) participants that received this intervention reported HIVST at least once and almost all (99%) reported not sharing their HIV self-tests with anyone. Roughly half (45%) of participants reported HIVST when they opened a new PrEP bottle and half (53%) reported that HIVST did not coincide with a specific event. 97% reported that using the self-test kit was easy or very easy. The majority (68%) of participants reported testing alone while some (31%) reported testing in the presence of their study partner. More than half (55%) reported that they did not share their test results with anyone.
Ortblad KF, *et al*., *BMC Infect Dis,* 2018 (NCT02827240 and NCT02846402)	Uganda and Zambia, Sept 2016–Oct 2016	FSW *N*=1382 Zambia (*N*=633); Uganda (*N*=749)	Cross-sectional survey at endline (among participants in two 1:1:1 randomized trials)	Values and preferences assessment: At the end of participation in an HIVST randomized trial, participants were asked (1) if they were interested in daily oral PrEP (5-point Likert scale) and (2) if they would prefer standard clinic-based HIV testing or HIVST while taking PrEP. Preferences for testing were evaluated across randomization arms: (1) direct HIV self-test delivery, (2) delivery of a coupon, exchangeable for an HIV self-test at nearby clinics, or (3) standard HIV testing services.	PrEP acceptability: The majority of participants in Zambia (91%) and Uganda (66%) reported that they would be “very interested” in taking daily oral PrEP. Revealed preferences: The majority of participants perceived a preference for HIVST compared to standard HIV testing services while on PrEP (87% Zambia; 82% Uganda). A greater percentage of participants in the HIVST intervention arms reported a perceived preference for HIVST compared to standard HIV testing services and these differences were statistically significant (*p*=0.002) and Uganda (*p*<0.001).
Wanga, V*. et al.* *J Int AIDS Soc* 2020 **POWER**	Kenya, Feb 2019–Nov 2019	AGYW *N*=249 *N*=172 women were offered HIVST at 202 PrEP follow-up visits. *N*=148 women used SOC at 202 visits.	Case study	Intervention: AGYWs at family planning clinics for a PrEP follow-up visits were given the option to choose between provider-initiated HIV testing or HIVST for PrEP continuation. Values and preferences assessment: *Revealed preferences* were measured by participants’ selection of their preferred testing option. *Feasibility* was assessed by determining how accurately participants completed the HIVST process.	Revealed preferences: Participants chose HIVST over provider-initiated HIV testing at about one-third of PrEP follow-up visits (35%, 70/202 visits). Among participants that selected HIVST, 96% said they would select it again in the future. Older age, never being married, and more PrEP follow-up visits were associated with selection of HIVST. Personal empowerment/taking charge of one’s health was the main reason for considering future HIVST and increased privacy/confidentiality was what most liked about HIVST. Feasibility: Clinic-based HIVST to support PrEP continuation is feasible; almost all participants that selected HIVST successfully completed the test (97%) and correctly read the test results (94%). HIVST also reduced the overall time spent in the clinic. Barriers: The main reason participants did not select HIVST for PrEP continuation was not feeling comfortable with testing alone (33% of visits), while the main reason they selected provider-initiated HIV testing was for counseling during testing (63% of visits). Satisfaction: Participants that selected HIVST for PrEP follow-up were more likely to be very happy with their overall testing experience compared to those that declined HIVST (73% vs. 47% of visits, *p* = 0.003). Almost all women rated their overall clinic experience as good (96% visits) and there were no statistically significant differences in satisfaction with the clinic experience among those who selected and did not select HIVST.

Abbreviations: *PrEP*, pre-exposure prophylaxis; *HIV*, human immunodeficiency virus; *HIVST*, HIV self-test; *SOC*, standard of care; *COVID-19*, coronavirus 2019; *SW*, sex workers; *FSWs*, female sex workers; *MSM*, men who have sex with men; *TGW*, transgender women; *TGNB*, transgender/non-binary; *AGYW*, adolescent girls and young women

^1^This table reports findings from 5 included articles of various study design

#### Acceptability

These various studies largely found that HIVST-supported models of PrEP delivery were acceptable in different settings and populations [23•, 24•, 27]. For example, in the Hoagland et al. study, most participants reported that PrEP teleconsultation was acceptable (70%), they preferred PrEP home delivery over clinic pick-up (87%), and they were willing to use an HIV self-test (79%) [23•]. In the Empower study, participants felt that HIVST between PrEP clinic visits empowered them economically (by testing clients for HIV and charging those who tested negative more for condomless sex), relationally (by restoring trust and intimacy with their sexual partners), and sexually (by addressing barriers, such as stigma, associated with accessing sexual health services and encouraging behaviors that prevent HIV risk acquisition, e.g., condom use) [27]. And finally, in the Partners Demonstration Project sub-study, HIVST between PrEP clinic visits reduced anxiety among participants while waiting to return for a PrEP clinic visit [24•].

#### Feasibility

Many of these studies also demonstrated that HIVST-supported models of PrEP delivery were feasible to implement [24•, 26•]. In the Partners Demonstration Project sub-study, almost all participants reported HIVST at least once between PrEP clinic visits (93%) – roughly half before a new PrEP bottle (45%) and half separate from a PrEP-related event (53%) – and that HIVST was easy (97%) [24•]. Additionally, in the POWER study, almost all participants that selected HIVST for PrEP continuation at family planning clinics successfully completed the HIV self-test (97%) and correctly read the HIV self-test result (94%); this intervention also reduced the overall time participants spent at their PrEP follow-up visit [26•].

#### Revealed Preferences

In the studies that measured revealed preferences, HIVST-supported models of PrEP delivery were preferred compared to other clinic-based or provider-delivered models [25•, 26•]. In the Ortblad et al. study, most participants (>80% in Zambia and Uganda) perceived a preference for HIVST versus clinic-based HIV testing while taking PrEP, with the participants in the HIVST intervention arms of two recently completed RCTs reporting a significantly greater preference for HIVST-supported PrEP continuation [25•]. In the POWER study, roughly a third of participants chose HIVST over provider-initiated HIV testing for PrEP continuation at family planning clinics and among those that selected HIVST, almost all (96%) said they would select it again in the future; older age, never being married, and more PrEP follow-up visits were associated with selection of HIVST [26•].

### Evidence Summary

We summarize the key findings from this systematic literature review, including risk of bias (in Table 4). We found that the overall risk of bias across all included studies was low to moderate (Appendix Figs. 1–4). For the three RCTs, most bias was attributable to missing outcomes and for the five case studies, bias was primarily attributable to confounding and selection of participants. In this table, we clarify that no studies were identified that explored HIVST use for PrEP initiation or for stopping and restarting PrEP.

**Table 4:** Summary of evidence on HIVST use to support PrEP delivery

Factor	Explanation/evidence
**Risk of bias**^**1**^	Low to moderate risk of bias.
**Balance of benefits vs. harms**	HIVST-supported models of PrEP delivery resulted in similar PrEP continuation outcomes to standard-of-care PrEP delivery with no adverse events.
**Effectiveness**	Adherence: HIVST-supported PrEP delivery models achieve similar (no effect/non-inferior effect) adherence compared to standard clinic-based PrEP delivery (Empower & JiPime-JiPrEP). HIVST use for PrEP continuation may increase adherence among single women (JiPime-JiPrEP) or as part of an adherence promotion package with biofeedback (PrEP-PP). Retention: HIVST-supported PrEP continuation achieves similar levels of PrEP retention and refills (no effect/non-inferior effect) compared to standard clinic-based PrEP delivery (Empower & JiPime-JiPrEP). HIV testing: HIVST use during PrEP continuation was high when used for interim or as prescribed testing in all three RCTs. HIV incidence: HIV incidence was very low across all three RCTs with only one person who seroconverted. Social harms: No adverse events or harms reported by using HIVST for PrEP continuation.
**Values and preferences**	Feasibility. Participants completed HIVST between PrEP clinic visits and returned to the clinic for scheduled follow-up. These models reduced the overall number of PrEP clinic visits and saved time. Acceptability. HIVST-supported models of PrEP delivery reduced anxiety associated with PrEP clinic visits and were preferred to clinic-based or provider-delivered PrEP.
**Economics/costs**	No cost data on models of PrEP delivery supported by HIVST were identified.
**Equity**	HIVST has the potential to increase equity by enabling more people to access or continue using PrEP.
**Research gaps**	There are no studies on HIVST use for PrEP initiation or for stopping and restarting PrEP. More evidence is needed in this research area to better understand if individuals interested in PrEP can safely initiate or re-initiate PrEP as needed themselves with the assistance of HIVST. Additionally, no information on the costs of these models is available at the moment.
^1^Risk of bias was measured using RoB2 for RCTs and ROBINS-I for non-RCTs	

## Discussion

This systematic review found that HIVST use for PrEP continuation results in similar adherence and retention outcomes compared to standard-of-care PrEP delivery. In addition, the use of HIV self-tests was perceived to be feasible and acceptable. Most of the included studies were conducted in sub-Saharan Africa and across different populations. In the RCTs, HIVST was used to support PrEP continuation as desired with testing between scheduled clinic visits [17••, 19••], and as prescribed with testing in lieu of scheduled visits [18••]. From these trials, we found that HIVST-supported models of PrEP delivery may increase adherence when delivered as part of an adherence package with biofeedback [19••] or may result in non-inferior adherence when used as a replacement for clinic visits [18••], compared to standard-of-care quarterly clinic-based PrEP delivery. None of the studies in this review directly evaluated the use of HIVST to support PrEP initiation.

This review highlights evidence for HIVST may be best used to simplify models of PrEP continuation rather than enhance existing models. For example, in the Empower study, where HIVST was additionally delivered with PrEP at scheduled quarterly clinic visits, this did not significantly impact PrEP continuation (e.g., retention and adherence) [17••]. And while a significant increase in PrEP adherence was observed in the PrEP-PP study, which tested an enhanced model of PrEP delivery that included HIVST, this effect was relatively modest and it was difficult to determine how much of the effect was attributable to HIVST delivery because the model also included adherence biofeedback and counseling [19••]. While this did add costs and complexity to some aspects of PrEP delivery, the ability for HIVST with counseling and adherence support to increase the number of people taking PrEP and reduce HIV incidence may be worth the investment. In contrast, a simplified model of PrEP delivery that reduced the number of clinic-based PrEP visits in half by replacing quarterly clinic-based testing with at-home HIVST, as tested in the JiPime-JiPrEP trial, resulted in non-inferior PrEP continuation (e.g., HIV testing, PrEP refilling, and adherence) compared to standard-of-care delivery [18••] with potential cost and time savings to both PrEP clients and providers (the costing analysis for this trial is forthcoming).

HIVST-supported models of PrEP delivery reflect a movement towards self-care and community-based care that may have benefits beyond just PrEP initiation and continuation. For example, if these models are used to simplify PrEP delivery, like in the JiPime-JiPrEP trial [18••], this could result in health systems savings and reduce the client burden associated with PrEP continuation. Additionally, access to HIVST in between PrEP refills could have psychological and emotional benefits, including feelings of empowerment associated with knowledge of HIV status and ability to control their HIV risk by overcoming physical and mental barriers to intimacy [27], as well as motivation to use PrEP to remain HIV uninfected [26•]. Such benefits could be particularly useful in populations with notably low adherence, such as adolescent girls and young women [28–30]. And while it was not explored explicitly in any of the studies identified in this review, PrEP clients with fluctuating HIV risk could potentially use HIV self-tests to stop and restart PrEP as needed with the appropriate guidance and instruction. Finally, in the era of COVID-19 and other potential future pandemics, HIVST-supported models of PrEP delivery could help decongest clinics, allowing healthcare provider to focus on serving sick patients, and mitigating potential disruptions to PrEP delivery [11].

In this review, we did not identify any completed, ongoing, or planned studies evaluating the use of HIVST to support PrEP initiation. We believe this is primarily attributed to two factors: (1) in many settings, PrEP still requires a prescription from a certified healthcare provider [31], and (2) concerns still remain around the sensitivity and specificity of HIV self-test kits, especially when it comes to diagnosing acute HIV infections [32]. First, if a healthcare provider is at risk of losing their license, they may be unmotivated to provide PrEP based on clients’ self-reported HIVST results. However, new online models of PrEP delivery are in development that may help address this by enabling individuals self-testing at home to upload an image of their self-test results, which providers can interpret and validate with the help of artificial intelligence algorithms [33], similar to models of COVID-19 testing that have been developed during the pandemic [34, 35]. Second, because HIV self-tests may detect HIV later than standard facility-based testing algorithms used for PrEP prescribing in many settings [36], concerns remain that someone living with HIV might be incorrectly diagnosed as HIV-negative and prescribed PrEP, which could lead to potential drug resistance [37]. Ongoing modelling studies suggest, however, that because acute and breakthrough HIV infections while using PrEP are rare, population-level PrEP drug resistance likely remains similar with or without HIVST use to support PrEP delivery. As a result, the potential benefit of HIVST enabling more people to access PrEP and avert new infections may outweigh likely this potential low-level risks of drug resistance [38].

The review had limitations that are important to note. First, the design of HIVST-supported models of PrEP delivery varied across the included studies, some using HIVST to support PrEP continuation as desired or as prescribed and some including HIVST as part of an intervention package with other things (e.g., adherence biofeedback). These differences made it difficult to draw direct comparisons between the studies and at times determine the effect that HIV self-test delivery alone had on study outcomes. Second, the measurement and timing of study outcomes differed across the included studies. Some studies measured PrEP adherence (i.e., TFV levels) using intracellular blood samples, while others used urine samples and some studies measured PrEP adherence at one month, while others measured it at six and 12 months, which again made comparisons across studies challenging. Third, feasibility and acceptability are multifaceted concepts for which the field of implementation research has yet to establish standard, validated metrics and definitions; thus, we captured diverse assessments for these outcomes in the review. Fourth, the review was limited to HIVST use for PrEP initiation and continuation and did not include the many interventions that have used HIVST to support linkage to PrEP services [39] or facilitate HIV testing among sexual partners [40–42] or peers [43]. Fifth, we only included English and peer-reviewed publications, and thus may have missed implementation projects that have used HIVST to support PrEP delivery but have not published these findings. Finally, most of the included studies took place among HIV priority populations in sub-Saharan Africa, which may limit the generalizability of the findings to other populations and settings.

## Conclusion

The existing evidence on HIVST-supported models of PrEP delivery is limited but suggests that HIVST has the potential to simplify PrEP delivery and that HIVST-supported delivery models are acceptable and feasible among diverse populations. It is possible that research on these models is limited because the WHO [11] and many countries [44, 45] currently do not recommend HIVST use for PrEP initiation and continuation outside of temporary adaptations to maintain essential services during the COVID-19 pandemic. Researchers should work in collaboration with policy makers, regulators, and implementors, to better understand their concerns around HIVST-supported models of PrEP delivery so they can generate evidence that fills these important research gaps and can be used to guide implementation studies and inform policy. In the HIV prevention world, there is a growing recognition that differentiated service delivery models can help decentralize prevention services and reach populations not engaged in traditional care models. Countries should consider integrating HIVST into PrEP delivery to potentially increase the resilience of service delivery and patient-centered care during an era of pandemics and achieve and maintain global HIV prevention targets.

## Supplementary Information

ESM 1 (DOCX 50 kb)

ESM 2 (DOCX 596 kb)

## Declarations

Not applicable as this was a review of published literature.
