# Supplementary material for: Examining the Use of HIV Self-Testing to Support PrEP Delivery: a Systematic Literature Review
Source: Curr HIV/AIDS Rep. 2022 Jul 29;19(5):394–408. doi: 10.1007/s11904-022-00617-x (PMC9334974; doi:10.1007/s11904-022-00617-x)
Supplement: Supplementary file 1 — (DOCX 50 kb) [file 11904_2022_617_MOESM1_ESM.docx]

**HIVST to support PrEP delivery: Systematic review**

PROTOCOL

Catherine Kiptinness^1^, Alexandra Kuo^2^, Adriana Reedy^3^, Cheryl Johnson^4^, Anjuli Wagner^5^, Katrina F. Ortblad^3^

^1^University of Nairobi, Kenya

^2^Department of Pharmacy, University of Washington, USA

^3^Public Health Sciences, Fred Hutchinson Cancer Research Center, USA

^4^World Health Organization, Geneva, Switzerland

^5^Department of Global Health, University of Washington, USA

**Background**

This systematic review will inform World Health Organization (WHO) guidelines on whether HIV self-testing (HIVST) can be used to support pre-exposure prophylaxis (PrEP) delivery (initiation and/or continuation). In our primary review, we will include effectiveness studies (i.e., randomized trials and quasi-experimental studies) and case studies that explore the use of HIVST in addition to and compared with existing HIV testing services (HTS) used to support PrEP delivery. Values and preferences of users and providers, and the costs and cost-effectiveness of using HIVST for PrEP delivery will be summarized descriptively. The review will also provide important information for mathematical modeling, particularly to determine clinical utility and risk-benefits of HIVST and standard HTS approaches which may vary by different product, programmatic implementation and testing time points and frequency of testing. This protocol provides a summary of the scope, definitions, and procedures that will be used in this review.

**Definitions**

HIV self-testing: A process where an individual performs a HIV test using either oral fluid or capillary (e.g., fingerstick whole blood) and then interprets their results. This can be done assisted or unassisted. All reactive self-tests need further testing to provide a definitive HIV-positive diagnosis. Nonreactive (negative) self-tests are accepted as negative and information on retesting and window period is provided to individuals.

PrEP initiation: The process of prescription and dispensing of PrEP for the first time or after a long break (i.e. re-starting).

PrEP continuation: The PrEP refill visits and continued use after PrEP initiation. This can include taking PrEP:

- *as prescribed* (e.g., every 3 months – both linked and not necessarily linked to PrEP refills)
- *as needed* (e.g., for stopping and restarting)
- *as desired* (e.g., between visits/refills)

We will include studies that report on the use of HIVST for PrEP delivery. This may include studies that assess HIVST in place of HTS or in addition to HTS at different time points. We will adhere to GRADE methodology.

**PICO question**

***Should HIVST be used to support PrEP delivery (including initiation and/or continuation)?***

| **P (Population)**: | Individuals at risk of HIV acquisition that could benefit from PrEP use |
| --- | --- |
| **I (Intervention):** | Utilization of HIVST kits for PrEP initiation, continuation and/or monitoring (in addition to or instead of HTS) |
| **C (Comparator):** | Standard HIV testing services (as defined in the study protocol) for PrEP initiation, continuation and/or monitoring without HIVST (delivered at clinics or in community) |
| **O (Outcomes)**: | Listed below |

***Primary outcomes***

1. PrEP initiation among participants (e.g., number of participants initiating/re-starting PrEP in study period among those screened or enrolled and randomized – depends on study design)
2. PrEP continuation among participants, including:
   1. Continued engagement with PrEP services (e.g., number of participants who continuously engaged with PrEP services in study period)
   2. Adherence (e.g., self-reported PrEP use, detectable drug levels in blood/hair/urine, pill counts)
   3. Testing *as prescribed* at 3-month intervals (*often, but not always, linked to PrEP refills)*
   4. Testing as *needed (e.g., for stopping and restarting PrEP)*
   5. *Testing as desired (e.g., between PrEP visits/refills)*

***Secondary outcomes****

1. HIV incidence among participants
2. HIVST performance (e.g., number of acute infections missed or number of false negatives compared to the standard-of-care testing method)
3. Drug resistance (e.g., number of cases of PrEP-related HIV mutations)
4. Sexual risk behavior (e.g., measured as report of condomless sex, sexual transmitted infections, or number of sexual partners).
5. HIV positivity (e.g., HIV-positive diagnosis among all participants initiating or restarting PrEP)
6. Social harms: number and proportion of testers who experienced social harm/adverse events. This includes physical, emotional, or sexual harms (e.g., violence from family members or community members, breach of confidentiality, psycho-social harm, self-harm, suicide, stigma, discrimination).

*Will only include secondary outcomes if a study reports on one of the primary outcomes above.

**Inclusion criteria**

To be included in the review, a study must meet the following criteria:

1. Study design* that compares a group, facility, geographic area, or population that used HIVST (instead of or in addition to HTS) to a group, facility, geographic area, or population that used only standard HTS without HIVST for PrEP (all forms included – e.g., daily oral, DPV, event-driven) initiation and/or continuation.
2. Measured one or more of the primary outcomes listed above.
3. Published in a peer-reviewed journal or conference abstract.

*If studies meet all the other criteria but do not present comparative data, then we will consider them as case studies. No restrictions will be placed on the intervention location. We only included English studies in the review.

**Search strategy**

The following databases, registries, and conferences will be searched:

- Electronic databases: PubMed, Embase, CINAHL Complete, Global Health Medicus, Cochrane CENTRAL (trials database), Web of Science, EconLit, PsycInfo, Social Serives, Abstracts, and the Global Health Database.
- Trial registries: Clinicaltrials.gov and the WHO International Clinical Trials Registry Platform.
- Conference abstracts: International AIDS Conference (AIDS), International AIDS Society Conference on HIV Science (IAS), International AIDS Society Conference on HIV Pathogenesis, Treatment, and Prevention, HIV Research for Prevention, and Retroviruses and Opportunistic Infections (CROI), and HIV Research for Prevention (HIVR4P). *Only abstracts available electronically will included.*

Secondary reference searching will be conducted on all studies included in the review. Selected experts in the field will be contacted to identify additional articles not identified through other search methods.

**Search terms**

Individual search strategies (for all databases, registries, and conferences described above) will be developed in collaboration with a University of Washington librarian based on the details in this protocol.

**Data extraction and management**

Data will be extracted independently by one reviewer using standardized data extraction forms. A secondary reviewer will do a non-blinded review of all extracted data. Differences in data extraction will be resolved through consensus.

The coding form will collect the following information from each included study:

- Study identification: Author(s); type of citation; year of publication
- Study description: Study objectives; location; population characteristics; study design; sample size; comparison groups; loss to follow-up
- Outcomes: Analytic approach (e.g., ITT or MITT); outcome measures; PrEP initiation / PrEP continuation / PrEP monitoring; count data; effect sizes; confidence intervals; significance levels; conclusions; limitations

**Stratifications**

We will report outcomes for the following categories:

- PrEP delivery: By PrEP initiation, PrEP continuation and PrEP monitoring
- Distribution of PrEP: facility-based vs. community-based vs. pharmacy-based vs. home-based
- HIVST type: blood-based vs. oral-fluid HIVST
- HIVST support: assisted HIVST vs. unassisted HIVST
- Distribution of HIVST: facility-based vs. community-based vs. pharmacy-based vs. home-based
- PrEP modality: e.g., daily oral, on demand, etc.
- Key and priority population: e.g., MSM, PWID, people in prisons and other closed settings, sex workers, transgender people, serodiscordant couples and adolescent girls and young women
- Sex/gender: cisgender/transgender men, cisgender/transgender women, non-binary individuals
- WHO region: African Region, Region of the Americas, South-East Asia Region, European Region, Eastern Mediterranean Region, and Western Pacific Region
- Income-level: low & lower-middle, upper-middle, and high-income countries

(World Bank classifications)

- Age groups:
- Ages ≤24 and ≥25 years
- Where available: 15-19, 20-24, 25-29, 30-34, 35-40 years

No restrictions will be placed based on location of the intervention.

**Values and preferences**

The same search terms will be used to search and screen for studies on the values and preferences of end users and providers. We seek to examine opinions, perspectives, values, and preferences related to PrEP initiation and continuation through the use of HIVST delivered at clinics or in the community. Studies reporting on linkage to PrEP and demand generation for PrEP using HIVST (see linkage to care section below) will be included if they report relevant findings on that values and preferences related to service delivery. Studies that include information on values and preferences could be qualitative or quantitative in nature, but have to present primary data collection (think pieces and review articles will not be included). Values and preferences literature will be summarized qualitatively and organized by study design, methodology, location, and population.

**Cost, cost-effectiveness and resource**

The same search terms will be used to search and screen for studies to be included in the cost review. Studies will be included in this review if they presented primary data on a number of related outcomes, including cost per PrEP initiation, costs per PrEP refill visit or a specified duration on PrEP, cost per new HIV infection averted among those using PrEP, and cost per disability-adjusted life year (DALY) averted or per quality adjusted life year (QALY) gained. Studies will also be included if they compare the costs, cost-effectiveness, cost-utility, or cost-benefit of models that use HIVST to support PrEP delivery with models that use traditional HIV testing services. Cost literature will be summarized qualitatively. Cost and resources may include personnel, HIVST kits, and program implementation cost.

**Linkage to care**

The same search terms will be used to search and screen for studies that use HIVST as a linkage to PrEP care intervention. These studies are unique because HIVST is not being used to inform PrEP prescribing or dispensing, rather just to bring people into PrEP care. In these studies, participants will still need to re-test for HIV using standard-of-care HIV testing tools for PrEP initiation (e.g., rapid diagnostic testing with PCR testing for confirmatory if necessary). These studies, which can include randomized trials, case studies, values and preferences, and costs, will be categorized as background studies. We only plan on including information on values and preferences or costs from these studies if they are related to using HIVST for PrEP initiation (not referral) or continuation.
