# Supplementary material for: Examining the Use of HIV Self-Testing to Support PrEP Delivery: a Systematic Literature Review
Source: Curr HIV/AIDS Rep. 2022 Jul 29;19(5):394–408. doi: 10.1007/s11904-022-00617-x (PMC9334974; doi:10.1007/s11904-022-00617-x)
Supplement: Supplementary file 2 — (DOCX 596 kb) [file 11904_2022_617_MOESM2_ESM.docx]

**Appendix Table 1. Description of study protocols on effectiveness and case studies related to HIVST use for PrEP delivery**, **N=4^1^**

| **Study** | **Country, Enrollment period** | **Population** | **Design** | **Intervention & outcome definition** | **Comparison** | **Effect on PrEP initiation and/or continuation** | **Other findings** |
| --- | --- | --- | --- | --- | --- | --- | --- |
| **Randomized controlled trials (N=3)** | | | | | | | |
| King R, et. al., *ClinicalTrials.gov* 2019  (NCT04030520)  **POPPi intervention** | Uganda,  July 2019-Oct 2021 | AGYW (N=60) | 1:1 randomized trial protocol  (parallel assignment) | Intervention: A package where participants receive a behavioral intervention, including counseling, and are offered PrEP as well as oral-fluid HIVST.  Primary outcomes: Initiation of PrEP (assessed using the pharmacy records) and PrEP adherence (measured via drug levels in hair samples) at 12 months. | SOC: A package where participants receive a behavioral intervention, including counseling, and are offered PrEP (but not oral-fluid HIVST). | Not reported: protocol paper | Not reported: protocol paper |
| Mugwanya K, et. al.,  *ClinicalTrials.gov* 2020  (NCT04424524)  **Efficiency Pilot** | Kenya,  May 2020-April 2022 | General population (N=500, either initiating or continuing PrEP) | Prospective observational study with comparator group protocol | Intervention: A pilot differentiated pharmacy based follow up PrEP care pathway. The core components of the multifaceted implementation strategy include: 1) 3-monthly refills, 2) direct-to-pharmacy refill visits, 3) HIV self-testing (HIVST) while waiting for refills, 4) rapid risk assessment for ongoing risk, adherence, side effect, and acute HIV symptoms.  Primary outcomes: PrEP wait time, PrEP continuation, PrEP adherence, acceptability & feasibility of direct-to-pharmacy, feasibility of direct-to-pharmacy care pathway.   - *Continuation*: Returned to the clinic for PrEP refill up to 6 months. - *Adherence:* TFV-DP levels in DBS sample collected at random PrEP clinic visits. | SOC: Clinics implementing current PrEP patient flow | Not reported: protocol paper | Not reported: protocol paper |
| Mujugira, A. et. al. *ClinicalTrials.gov 2020*  (NCT04328025)  **Peer Study** | Uganda, Oct 2020-June 2022 | TGW (N=82) | 1:1 randomized trial protocol  (parallel assignment) | Intervention: Peers will deliver HIVST, STI self-sampling and PrEP monthly, in between quarterly PrEP clinic visits, and deliver associated adherence counseling. Peers will also distribute STI self-sampling kits for personal use or sharing with regular partners. In addition, peers will also motivate ongoing adherence, promote repeat HIV testing, and support PrEP use as problems arise. Additionally, peers will remind TGW to self-test before opening a new PrEP bottle. They will present smartphone instructional videos showing how to self-collect specimens for STI testing.  Primary outcomes: PrEP adherence, as measured by intracellular TFV-DP levels in DBS samples at 12 months. | SOC: Quarterly clinic-based HIV counseling, PrEP dispensing (with RDT), and management of STIs per standard of care. | Not reported: protocol paper | Not reported: protocol paper |
| **Case studies (N=1)** | | | | | | | |
| El-Sadr WM, et. al., *ClinicalTrials.gov* 2022  (NCT04898699)  **IPrEP Men’s study** | Kenya,  Oct 2021-Mar 2022 | Male clients of FSW (N=120) | Case study: protocol | Intervention: HIVST at the discretion of the participant and prior to starting a new bottle of PrEP medication in between study visits.  Primary outcomes: PrEP adherence, as measured by plasma TFV concentration levels. | N/A | Not reported: protocol paper | Not reported: protocol paper |

**Abbreviations:** pre-exposure prophylaxis (PrEP); sexually transmitted infections (STIs); female sex workers (FSWs); men who have sex with men (MSM); transgender women (TGW); adolescent girls and young women (AGYW); standard of care (SOC); rapid diagnostic testing (RDT); dried blood spots (DBS); tenofovir diphosphate (TFV-DP)

^1^This section of the table reports findings from 6 study protocols (4 are randomized trial protocols, 2 are case study protocols), with 2 studies also reporting values and preferences.

**Appendix Table 2. Description of protocols exploring values and preferences and costs HIVST to support PrEP delivery, N=2^1^**

| **Study** | **Country, Enrollment period** | **Population** | **Study design** | **Intervention & outcome assessment** | **Key findings on preferences for HIVST-supported PrEP delivery** |
| --- | --- | --- | --- | --- | --- |
| **Values and preferences studies (N=2)** | | | | | |
| Mugwanya K, et. al.,  *ClinicalTrials.gov* 2020  (NCT04424524)  **Efficiency**  *(Also included in Appendix 1)* | Kenya,  May 2020-April 2022 | General population (N=500, either initiating or continuing PrEP) | Prospective observational study with comparator group protocol | Intervention: A prospective pilot evaluation of clinics that will implement either the current PrEP patient flow without any change or a pilot differentiated pharmacy-based follow up PrEP care pathway. The core components of the multifaceted implementation strategy include: 1) 3-monthly refills, 2) direct-to-pharmacy refill visits, 3) HIVST while waiting for refills, 4) Rapid risk assessment for ongoing risk, adherence, side effect, and acute HIV symptoms.  Values and preferences assessment: Quantitative data on PrEP users will be evaluated to understand delivery efficiency and in-depth interviews with users and key informants will be conducted to identify barriers and facilitators of implementation. The PrEP delivery services will be assessed via patient waiting time, early PrEP continuation, and PrEP adherence. A mixed methods study will be conducted to understand patient and provider perception, experiences, feasibility, and acceptability of a differentiated PrEP delivery model. The acceptability of HIVST use for PrEP delivery will also be assessed. The safety of HIVST and reasons for PrEP discontinuation will be reviewed. Cost of implementing direct-to-pharmacy PrEP care will be analyzed. | Not reported: protocol paper |
| Mujugira, A*. et. al. 2020*  (NCT04328025)  *(Also included in Appendix 1)* | Uganda,  Oct 2020-June 2022 | TGW (N=82) | 1:1 randomized trial protocol  (parallel assignment) | Intervention: Peers will deliver HIVST, STI self-sampling and PrEP monthly, in between quarterly PrEP clinic visits, and deliver associated adherence counseling. Peers will also distribute STI self-sampling kits for personal use or sharing with regular partners. In addition, peers will also motivate ongoing adherence, promote repeat HIV testing, and support PrEP use as problems arise. Additionally, peers will remind TGW to self-test before opening a new PrEP bottle. They will present smartphone instructional videos showing how to self-collect specimens for STI testing.  Values and preferences assessment: Quarterly clinic-based testing will confirm accuracy of self-tests and identify inaccurate test results. Feasibility of peer-delivery intervention will be assessed by the proportion of kits successfully delivered by peers at 12 months. Acceptability of intervention will be measured by surveys and qualitative interviews and by the proportion of participants using HIVST/STI self-testing kits at 12 months. | Not reported: protocol paper |

**Abbreviations:** pre-exposure prophylaxis (PrEP); human immunodeficiency virus (HIV); sexually transmitted infection (STI); HIV self-test (HIVST); men who have sex with men (MSM); transgender women (TGW); adolescent girls and young women (AGYW)

^1^This table reports findings from 2 included study protocols, all of which are also included in Appendix 1.


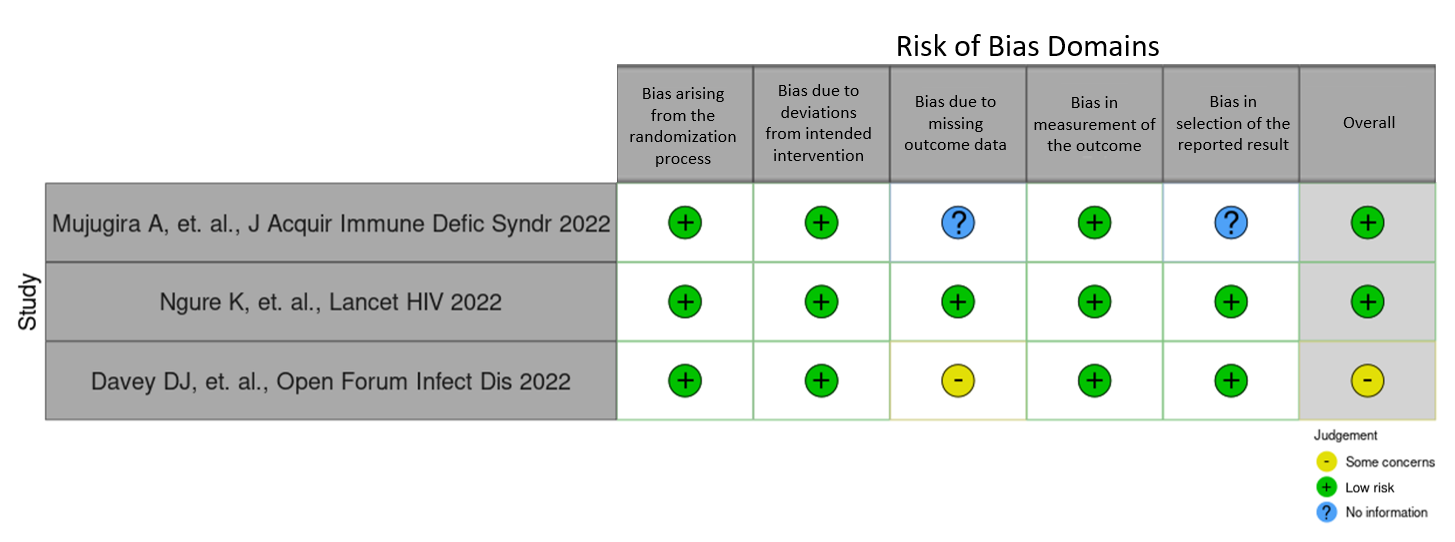


**Appendix Figure 1. Traffic light plot - quality assessment of extracted literature using ROB2**


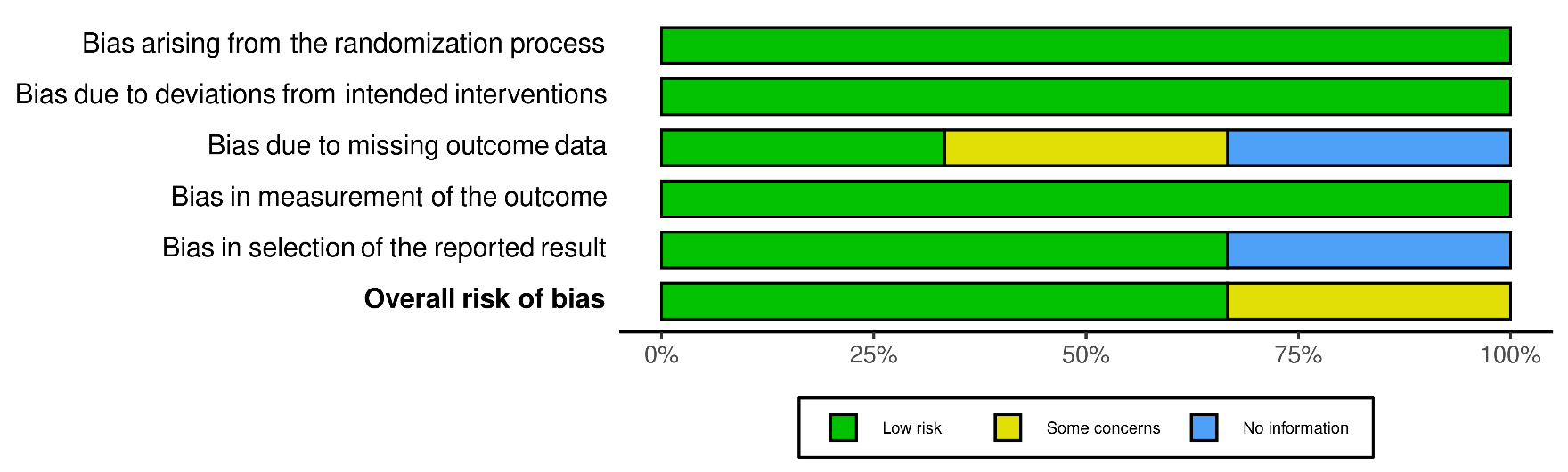


**Appendix Figure 2. Summary plot - quality assessment of extracted literature using ROB2**

**
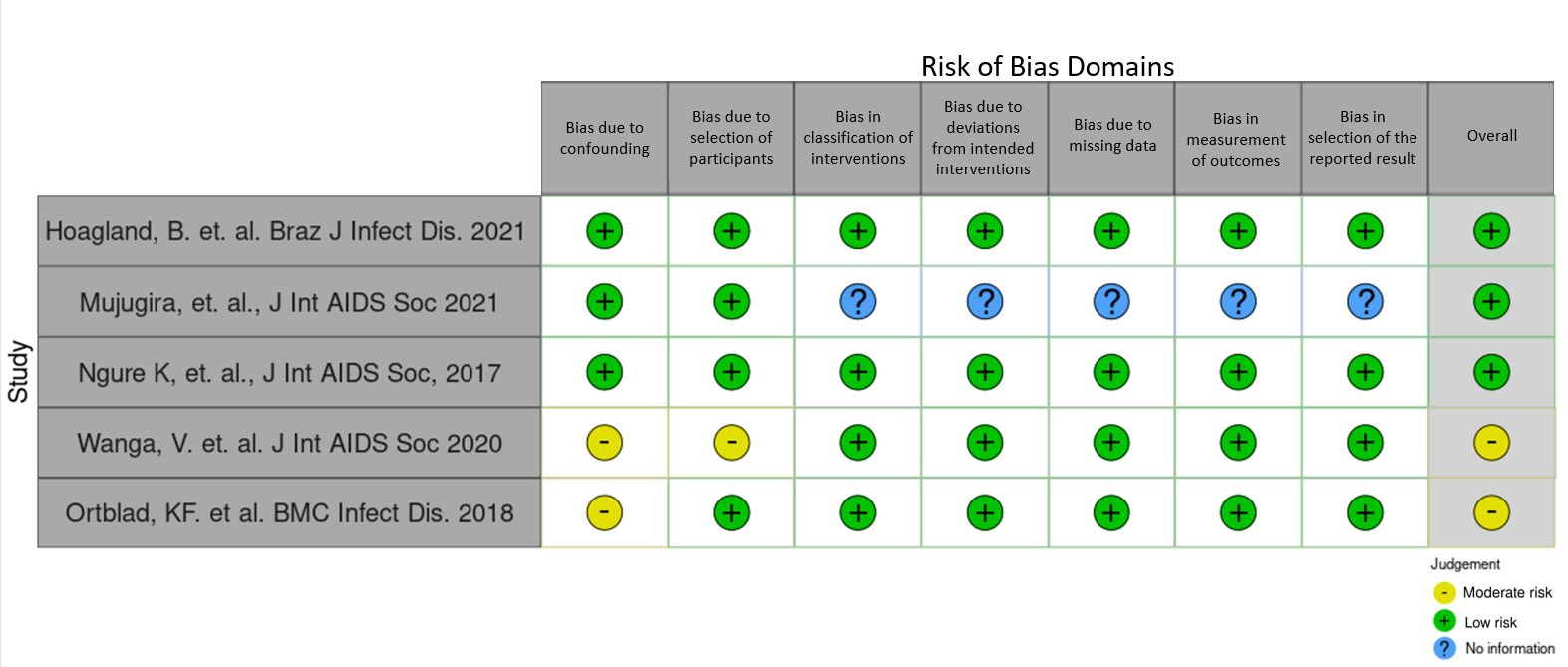
**

**Appendix Figure 3. Traffic light plot - quality assessment of extracted literature using ROBINS-I**


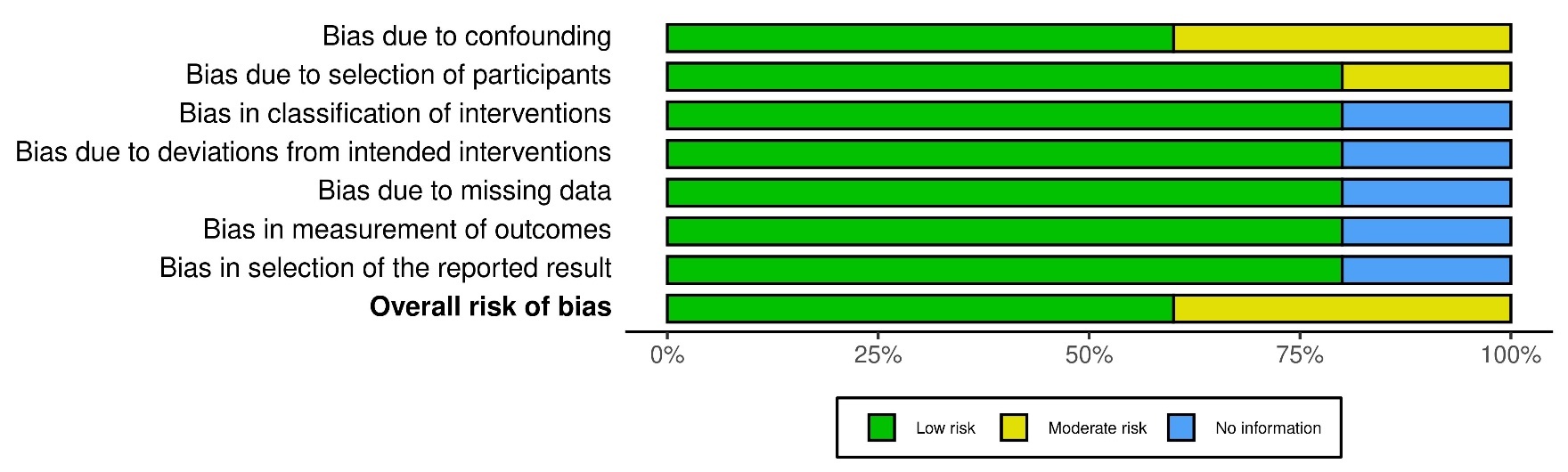


**Appendix Figure 4. Summary plot - quality assessment of extracted literature using ROBINS-I**
